# A woman dies every two minutes due to pregnancy or childbirth: UN agencies

**Published:** 2023-03

**23 February 2023** - Every two minutes, a woman dies during pregnancy or childbirth, according to the latest estimates released in a report by United Nations agencies today. This report, Trends in maternal mortality, reveals alarming setbacks for women’s health over recent years, as maternal deaths either increased or stagnated in nearly all regions of the world.

“While pregnancy should be a time of immense hope and a positive experience for all women, it is tragically still a shockingly dangerous experience for millions around the world who lack access to high quality, respectful health care,” said Dr Tedros Adhanom Ghebreyesus, Director-General of the World Health Organization (WHO). “These new statistics reveal the urgent need to ensure every woman and girl has access to critical health services before, during and after childbirth, and that they can fully exercise their reproductive rights.”

The report, which tracks maternal deaths nationally, regionally and globally from 2000 to 2020, shows there were an estimated 287 000 maternal deaths worldwide in 2020. This marks only a slight decrease from 309 000 in 2016 when the UN’s Sustainable Development Goals (SDGs) came into effect. While the report presents some significant progress in reducing maternal deaths between 2000 and 2015, gains largely stalled, or in some cases even reversed, after this point.

In two of the eight UN regions – Europe and Northern America, and Latin America and the Caribbean – the maternal mortality rate increased from 2016 to 2020, by 17% and 15% respectively. Elsewhere, the rate stagnated. The report notes, however, that progress is possible. For example, two regions – Australia and New Zealand, and Central and Southern Asia – experienced significant declines (by 35% and 16% respectively) in their maternal mortality rates during the same period, as did 31 countries across the world.

“For millions of families, the miracle of childbirth is marred by the tragedy of maternal deaths,” said UNICEF Executive Director Catherine Russell. “No mother should have to fear for her life while bringing a baby into the world, especially when the knowledge and tools to treat common complications exist. Equity in healthcare gives every mother, no matter who they are or where they are, a fair chance at a safe delivery and a healthy future with their family.”

In total numbers, maternal deaths continue to be largely concentrated in the poorest parts of the world and in countries affected by conflict. In 2020, about 70% of all maternal deaths were in sub-Saharan Africa. In nine countries facing severe humanitarian crises, maternal mortality rates were more than double the world average (551 maternal deaths per 100 000 live births, compared to 223 globally).

“This report provides yet another stark reminder of the urgent need to double down on our commitment to women and adolescent health,” said Juan Pablo Uribe, Global Director for Health, Nutrition and Population at the World Bank, and Director of the Global Financing Facility. “With immediate action, more investments in primary health care and stronger, more resilient health systems, we can save lives, improve health and well-being, and advance the rights of and opportunities for women and adolescents.”

Severe bleeding, high blood pressure, pregnancy-related infections, complications from unsafe abortion, and underlying conditions that can be aggravated by pregnancy (such as HIV/AIDS and malaria) are the leading causes of maternal deaths. These are all largely preventable and treatable with access to high-quality and respectful healthcare.

Community-centered primary health care can meet the needs of women, children and adolescents and enable equitable access to critical services such as assisted births and pre- and postnatal care, childhood vaccinations, nutrition and family planning. However, underfunding of primary health care systems, a lack of trained health care workers, and weak supply chains for medical products are threatening progress.

Roughly a third of women do not have even four of a recommended eight antenatal checks or receive essential postnatal care, while some 270 million women lack access to modern family planning methods. Exercising control over their reproductive health – particularly decisions about if and when to have children – is critical to ensure that women can plan and space childbearing and protect their health. Inequities related to income, education, race or ethnicity further increase risks for marginalized pregnant women, who have the least access to essential maternity care but are most likely to experience underlying health problems in pregnancy.

“It is unacceptable that so many women continue to die needlessly in pregnancy and childbirth. Over 280,000 fatalities in a single year is unconscionable,” said UNFPA Executive Director Dr. Natalia Kanem. “We can and must do better by urgently investing in family planning and filling the global shortage of 900,000 midwives so that every woman can get the lifesaving care she needs. We have the tools, knowledge and resources to end preventable maternal deaths; what we need now is the political will.”

The COVID-19 pandemic may have further held back progress on maternal health. Noting the current data series ends in 2020, more data will be needed to show the true impacts of the pandemic on maternal deaths. However, COVID-19 infections can increase risks during pregnancy, so countries should take action to ensure pregnant women and those planning pregnancies have access to COVID-19 vaccines and effective antenatal care.

“Reducing maternal mortality remains one of the most pressing global health challenges,” said John Wilmoth, Director of the Population Division of the Department of Economic and Social Affairs. “Ending preventable maternal deaths and providing universal access to quality maternal health care require sustained national and international efforts and unwavering commitments, particularly for the most vulnerable populations.  It is our collective responsibility to ensure that every mother, everywhere, survives childbirth, so that she and her children can thrive.”

The report reveals that the world must significantly accelerate progress to meet global targets for reducing maternal deaths, or else risk the lives of over 1 million more women by 2030.

Available from: https://www.who.int/news/item/23-02-2023-a-woman-dies-every-two-minutes-due-to-pregnancy-or-childbirth--un-agencies

